# Primary fallopian tube cancer followed by primary breast cancer in RAD51C mutation carrier treated with niraparib as first line maintenance therapy: a case report

**DOI:** 10.1186/s13053-024-00274-8

**Published:** 2024-02-15

**Authors:** Hua Yuan, Rong Zhang, Ning Li, Hongwen Yao

**Affiliations:** Department of Gynecologic Oncology, National Clinical Research Center for Cancer/Cancer Hospital, National Cancer Center, Chinese Academy of Medical Sciences and Peking Union Medical College, 17 # Panjiayuannanli, Chaoyang District, Beijing, 100021 P.R. China

**Keywords:** Primary breast cancer, Primary fallopian tube cancer, RAD51C mutation, Niraparib case report

## Abstract

Given the rarity of RAD51C mutations, the risk and treatment of metachronous breast cancer after the diagnosis of ovarian cancer in RAD51C mutation carriers is not clear, especially for those who have received PARPi treatment. We report the case of a 65-year-old woman diagnosed with stage IIIC high-grade serous primary fallopian tube cancer. The patient had no family history of breast or ovarian cancer. The patient received three cycles of neoadjuvant chemotherapy with paclitaxel and carboplatin and achieved a complete response. After interval debulking surgery, the patient received three cycles of adjuvant chemotherapy. Collection and extraction of saliva DNA for next-generation sequencing identified a RAD51C mutation c.838-2 A > G. The patient received niraparib as front-line maintenance treatment. After 36 months of niraparib treatment, the patient had grade II invasive ductal carcinoma of the left breast that was positive for estrogen receptor (90%) and Ki-67 (30%) and negative for progesterone receptor and human epidermal growth factor receptor 2. Computed tomography revealed the absence of distant metastases. Modified radical mastectomy and axillary lymph node dissection were then performed. The final pathological report of the breast showed a 1.8 cm Bloom-Richardson grade II invasive ductal carcinoma in the left breast with axillary lymph node metastasis (1/21). Finally, the breast cancer was stage IIA, pT1cN1M0. The metachronous breast cancer in this case may be the first report of second primary cancer in fallopian tube cancer patient harboring a RAD51C mutation during niraparib treatment. Further studies are required to determine optimal treatment.

## Introduction

RAD51 paralogues (RAD51B, RAD51C, RAD51D, XRCC2, and XRCC3) have recently been implicated in breast and ovarian cancer predisposition [1]. RAD51 family members are involved in homologous recombination and DNA repair. Germline mutations in breast and ovarian cancer pedigrees have established RAD51C as a human cancer susceptibility gene [2]. The frequency of RAD51C mutations was 0.41% (14/3429) in epithelial ovarian cancer (EOC) cases in a large case-control study [3]. The estimated cumulative risks of developing tubo-ovarian carcinoma (TOC) and breast cancer (BC) at 80 years of age were 11% (95% CI:6–21%) and 21% (95% CI:15–29%) for RAD51C, respectively [4]. Clinical trials have demonstrated promising response rates among patients with ovarian cancer receiving poly (ADP-ribose) polymerase (PARP) inhibitors, especially for those with BRCA1/2 germline mutations. RAD51C and RAD51D mutations can predict the response to PARP inhibitors, similar to BRCA1/2 mutations [5]. 

The number of diagnoses of secondary breast cancer after ovarian cancer has decreased [6]. Few studies have evaluated the risk of breast cancer after diagnosis of EOC in patients with BRCA1/2 mutations [7, 8]. Given the rarity of RAD51C mutations, the risk of breast cancer after a diagnosis of EOC in RAD51C mutation carriers is not clear. Moreover, there are no established treatment guidelines for these cases, especially for those who have received PARPi treatment.

Here, we present a rare case of primary breast cancer following primary fallopian tube cancer in a RAD51C mutation carrier, treated with niraparib. To the best of our knowledge, the metachronous breast cancer in this case may be the first report of second primary cancer in a fallopian tube cancer patient harboring a RAD51C mutation during niraparib treatment.

## Case presentation

**Fig. 1:**
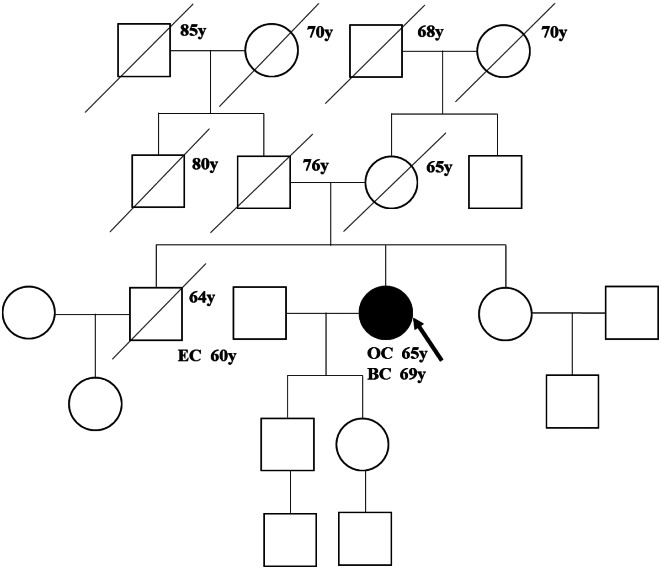
Pedigree including first-, and second-degree relatives of the patient with RAD51C: c.838-2 A > G. Abbreviations: OC, ovarian cancer; BC, breast cancer; EC, esophageal cancer

A 65-year-old female patient presented to our hospital with abdominal pain and anorexia. The patient’s medical history included hypertension. We collected the detailed family information. All information of first- and second-degree relatives were verified. Pedigree including first-, and second-degree relatives of this patient was shown in Fig. 1. The family history revealed that her brother had esophageal cancer. The patient had no family history of breast or ovarian cancers. Her height was 154 cm and she weighed 63 kg. Physical examination revealed a distended abdomen and a palpable 4.0 × 4.0 cm solid mass in the left adnexal area with poor mobility. An initial computed tomography (CT) scan of the abdomen and pelvis revealed a 4.1 × 3.6 cm left pelvic mass, thickened greater omentum, mesentery, and peritoneum with variable nodules and a large amount of ascites. The serum cancer antigen-125 (CA-125) levels were 352.4 U/mL. Simple laparoscopic surgical exploration was performed. Macroscopic findings showed a left adnexal tumor measuring 5.0 × 4.0 cm. Bilateral salpingectomy was performed, and suspected disseminated nodules on the mesorectal surface were resected. Histopathological examination of the excised specimens showed that the left adnexal tumor was a papillary solid mass, and the histological type was a primary high-grade serous carcinoma of the fallopian tube.

Neoadjuvant chemotherapy (NAC) with paclitaxel (175 mg/m^2^) and carboplatin (AUC = 5) was administered every three weeks. CA125 levels normalized after three cycles of NAC. After the completion of three cycles of NAC treatment, the patient underwent total hysterectomy, bilateral oophorectomy, omentectomy, and pelvic lymph node dissection. In addition, the suspected disseminated nodules of the pelvic peritoneum were resected, with no gross residual disease. The second histopathological examination of excised specimens showed a complete pathological response to NAC. The final stage was stage IIIC, high-grade serous primary fallopian tube cancer. Adjuvant chemotherapy consisting of paclitaxel (175 mg/m^2^) and carboplatin (AUC = 5) was administered. Collection and extraction of saliva DNA for next-generation sequencing identified a RAD51C mutation c.838-2 A > G (NM_058216.3, Chr17:58720744) in germline DNA. The patient received niraparib as front-line maintenance treatment after adjuvant chemotherapy. A laboratory test and imaging evaluation were performed every three months during the PARPi treatment and showed no signs of recurrence and a normal level of CA125.

**Fig. 2:**
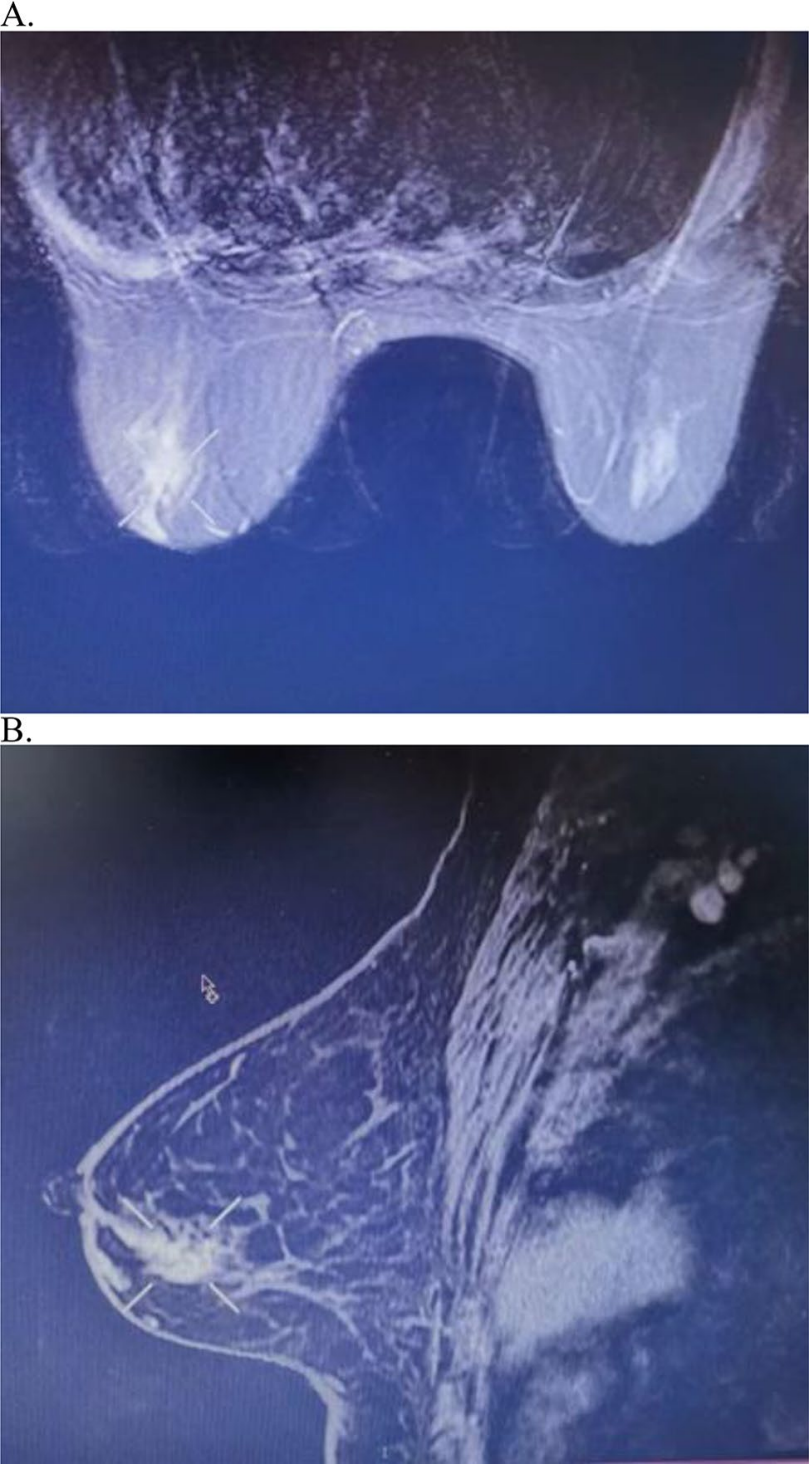
MRI scanning of the left breast

36 months after niraparib treatment, the patient noticed a palpable mass in the left breast. The mass in the left breast and presumed regional lymph nodes in the left axilla were palpable on physical examination. Mammography revealed fine pleomorphic calcifications in the left breast. There was a 1.1 cm × 3.0 cm sized irregular, segmental contrast-enhanced mass in the lower quadrant of the left breast on magnetic resonance imaging (MRI). (Fig. 2) A core needle biopsy of the suspicious breast and left axillary masses was performed under ultrasound (US) guidance. Biopsy results of the breast lesion revealed grade II invasive ductal carcinoma (IDC), which was positive for estrogen receptor (ER, 90%) and Ki-67 (30%), and negative for progesterone receptor (PR) and human epidermal growth factor receptor 2 (HER2). Contrast-enhanced chest, abdominal, and pelvic computed tomography (CT) revealed no distant metastases.

The patient showed no signs of recurrence of TOC. After the diagnosis of breast cancer, the patient discontinued niraparib treatment. Modified radical mastectomy (MRM) and left axillary lymph node dissection (ALND) was performed. The final pathological report showed a 1.8 cm Bloom-Richardson grade II invasive ductal carcinoma in the left breast with left axillary lymph node metastasis (1/21). Immunohistochemical analysis of the breast lesions revealed the expression of ER (95%) and PR (3%), but not HER2, with a Ki-67 rate of 30%. Finally, the metachronous breast cancer in this patient was staged as stage IIA pT1cN1M0.

**Fig. 3:**
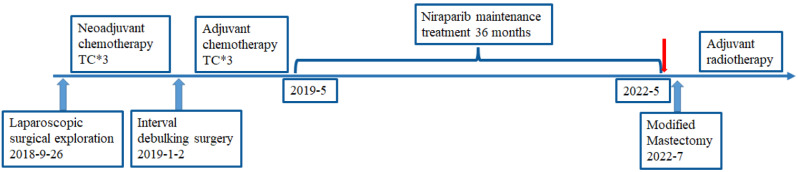
Time line of the therapeutic process

The patient refused chemotherapy. Subsequently, a multidisciplinary discussion was conducted, following which radiotherapy was chosen. The patient underwent adjuvant radiotherapy at another center without PARP inhibitor treatment. The therapeutic process was showed in Fig. 3.

## Discussion and conclusions

RAD51C germline mutation carriers have a higher tendency to develop TOC and BC. However, the risk of breast cancer after a diagnosis of TOC in RAD51C mutation carriers remains unclear. The patient in the present study received niraparib maintenance treatment after first-line chemotherapy and survived for 3 years with no signs of TOC recurrence, and metachronous breast cancer was diagnosed at an early stage.

The c.838-2 A > G intronic variant results from an A to G substitution two nucleotides upstream of exon 6 in RAD51C [2, 9]. The mutation is expected to disrupt RNA splicing and likely results in an absent or disrupted protein product. The mutation is classified as likely pathogenic in ClinVar. However, the actual pathogenicity of this mutation is unknown. Functional analysis of this mutation should be performed in the future. Individuals with RAD51C mutations have a higher risk of developing primary breast or ovarian cancer than non-carriers. Both TOC and BC risks for RAD51C pathogenic mutation carriers vary according to family history of cancer [4]. Carriers of a germline mutation in any of the RAD51 genes were more likely to have a family history of ovarian cancer, although the difference was not statistically significant [3]. However, no family history was found in the present patient.

Ovarian cancer is the most common cause of death among gynecological cancers. Women with ovarian cancer who had secondary breast cancer had superior cause-specific survival compared to those who did not develop breast cancer, regardless of breast cancer timing [6]. Clinical trials have demonstrated promising response rates among ovarian cancer patients with BRCA1/2 germline mutations treated with PARP inhibitors. RAD51C and RAD51D mutations can also predict the response to PARPis, similar to BRCA1/2 mutations [5]. Our present observations are in agreement with these findings. In the present study, the patient tended to benefit from niraparib treatment. Studies have shown that mutations in other homologous recombination genes have a similar positive impact on overall survival and platinum responsiveness to germline BRCA1/2 mutations [10]. Overall, the exact prognostic role of RAD51C mutation should be determined in multicenter studies with larger sample sizes.

Interestingly, Kondrashova et al. found that analyses of primary and secondary mutations in RAD51C and RAD51D could provide evidence for these primary mutations conferring PARPi sensitivity and secondary mutations as a mechanism of acquired PARPi resistance [11]. PARPi resistance due to secondary mutations underpins the need for early delivery of PARPi therapy and combination strategies. With the occurrence of breast cancer, whether the patient have developed resistance to PARPi was not sure since there were no signs of recurrence of ovarian cancer.

Emerging evidence has found that patients with BRCA-associated OC have a lower risk of developing subsequent primary or contralateral breast cancer than mutation carriers without OC [12]. For patients with BRCA-associated ovarian cancer, overall survival is dominated by OC deaths, and the risk of dying from OC is greater than the risk of developing BC. These results support nonsurgical management of BC risk in women with BRCA-OC [7]. Imaging surveillance should be advocated during the first several years after ovarian cancer diagnosis, after which the benefits of risk-reducing mastectomy (RRM) can be considered based on patient age and BRCA mutation status [13]. Some researchers have suggested that RRM or MRI screening should only be recommended for those who have survived ovarian cancer without recurrence for ten years and for those with early stage ovarian cancer [14]. The planned screening and prevention strategies for breast cancer have not been determined for ovarian cancer patients with RAD51C mutations. Intensive breast screening was performed in the present patient but not for preventive mastectomy. It is worth noting that multi-omics analysis and liquid biopsy should be performed to identify the underlying mechanisms of metachronous breast cancer.

In the era of PARPi, PARPi can prevent breast cancer occurrence but also prolong the survival of patients with ovarian cancer and increase the possibility of breast cancer occurrence. The risk assessment and screening strategy for metachronous breast cancer in ovarian cancer patients with RAD51C mutations should be determined by incorporating many factors such as family history, mutation status, PARPi response, tumor stage, and survival. The age at diagnosis of ovarian cancer may be higher in patients with mutations in RAD51. Song et al. found that more RAD51 mutation carriers were diagnosed at ages 40–49 years than noncarriers, and no mutation carrier were diagnosed with ovarian cancer before the age of 40 years [3]. The age at diagnosis of ovarian cancer in the present case was 65 years. Since the age of onset of breast and ovarian cancer in RAD51C mutation carriers is older, intensive breast screening as well as oophorectomy should be implemented even after the age of 60 for RAD51C mutation carriers. Most breast cancers following ovarian cancer at an early stage are detected by mammography in BRCA1/2 mutation carriers [8]. Average 3.3 years of subsequent breast cancer are diagnosed after EOC in BRCA carriers. The interval between TOC and BC diagnosis was 3.5 years in the present case, which is in accordance with previous studies. our case, metachronous breast cancer occurred following primary ovarian cancer in a RAD51C mutation carrier during niraparib treatment. Given the rarity of RAD51C mutations, there are no established treatment guidelines for these cases, especially for those who received PARPi treatment. Patients should receive adjuvant chemotherapy according to the pathological findings of metachronous breast cancer. Based on the results of the OlympiAD and EMBRACA trials, the two PARP inhibitors, olaparib and talazoparib, are included as category 1 preferred options for recurrent or stage IV breast cancer with germline BRCA1/2 mutations [15]. Whether the patient in the present study could continue to receive niraparib or another PARPi treatment is not sure. Whether adjuvant chemotherapy, radiotherapy, and endocrine therapy could be combined with PARPi therapy, if required, has not been determined. The optimal treatment plan may need to be determined through multidisciplinary discussion.

To the best of our knowledge, the metachronous breast cancer in this case may be the first report of second primary cancer in a fallopian tube cancer patient harboring a RAD51C mutation during niraparib treatment. Further studies are needed to determine optimal treatment. As patients with TOC have a longer life expectancy in the era of PARPi, the risk of second primary cancer following TOC is worth our attention, especially for those with germline mutations in homologous recombination repair genes. It’s worth noting that breast cancer is a common disease in women with older age. The development of breast cancer in this patient may also be caused by other mechanisms. Loss of heterozygosity analysis of RAD51C in breast cancer should be performed to clarify the pathogenesis.

In conclusion, patients with RAD51C mutations tend to derive benefits from niraparib treatment. Primary breast cancer following primary fallopian tube cancer in RAD51C mutation carriers treated with niraparib is rare, and the optimal treatment plans for these patients need to be determined through multidisciplinary discussion.

## Data Availability

No datasets were generated or analysed during the current study.

## Abbreviations

EOC: epithelial ovarian cancer
TOC: Tubo-ovarian carcinoma
BC: Breast cancer
PARP: Poly (ADP-ribose) polymerase
CT: Computed tomography
CA-125: Cancer antigen-125
NAC: Neoadjuvant chemotherapy
MRI: Magnetic resonance imaging
ER: Estrogen receptor
PR: Progesterone receptor
HER2: Human epidermal growth factor receptor 2
RRM: risk-reducing mastectomy
